# 853. Real-World Persistency of Patients Receiving Tenofovir-Based Pre-Exposure Prophylaxis for the Prevention of HIV Infection in the US

**DOI:** 10.1093/ofid/ofab466.1048

**Published:** 2021-12-04

**Authors:** Alan Oglesby, Guillaume Germain, Francois Laliberte, Staci Bush, Heidi Swygard, Sean MacKnight, Annalise Hilts, Mei Sheng Duh

**Affiliations:** 1 ViiV Healthcare, Research Triangle Park, NC; 2 Analysis Group, Montreal, Quebec, Canada; 3 Analysis Group, Inc., MA

## Abstract

**Background:**

Once-daily oral tenofovir-based combinations as pre-exposure prophylaxis (PrEP) have shown to be an effective biomedical HIV prevention strategy for populations at-risk of acquiring HIV-1. However, low adherence can lead to poor effectiveness. This study described the characteristics of commercially-insured US PrEP users.

**Methods:**

This retrospective study used IQVIA™ PharMetrics Plus data (1/1/2015–3/31/2020) to identify adults newly initiated (index date) on emtricitabine/tenofovir disoproxil fumarate (FTC/TDF) or emtricitabine/tenofovir alafenamide (FTC/TAF) as daily PrEP. Users had ≥6 months of continuous enrollment pre-index (baseline); those diagnosed with HIV or with antiretroviral therapy (ART) use during baseline were excluded. User characteristics were described during the baseline period. For FTC/TDF users, proportion of days covered (PDC), persistence, treatment breaks, and switching were described during the follow-up period, which spanned from index to the earliest of disenrollment or end of data. Non-persistence was defined as a >90-day gap from last day of supply, with re-initiation after this gap indicating treatment break. For PDC and persistence, follow-up was censored at HIV infection, defined by both multi-class ART initiation and HIV diagnosis.

**Results:**

In total 24,232 FTC/TDF and 1,187 FTC/TAF users were identified. Overall, mean age was 35.1 years and 94.5% were male (Table 1). Mean [median] length of follow-up was longer for FTC/TDF (504 [390] days) than FTC/TDF users (77 [70] days). On average, FTC/TDF users had 9.0 dispensings with 38.3 days of supply per dispensing over follow-up; 11.1% had ≥1 treatment break (mean length, 249 days). Among those initiated on FTC/TDF, 10.8% switched to FTC/TAF. The mean PDC for FTC/TDF users at 6 and 12 months was 0.74 and 0.67, respectively, corresponding to 63.7% and 57.9% of patients with PDC ≥0.70 (Figure 1). Persistence to FTC/TDF at 6 and 12 months was 70.2% and 57.4%, respectively (Figure 2).

Table 1. Baseline Demographics and Clinical Characteristics of PrEP Users by Regimen

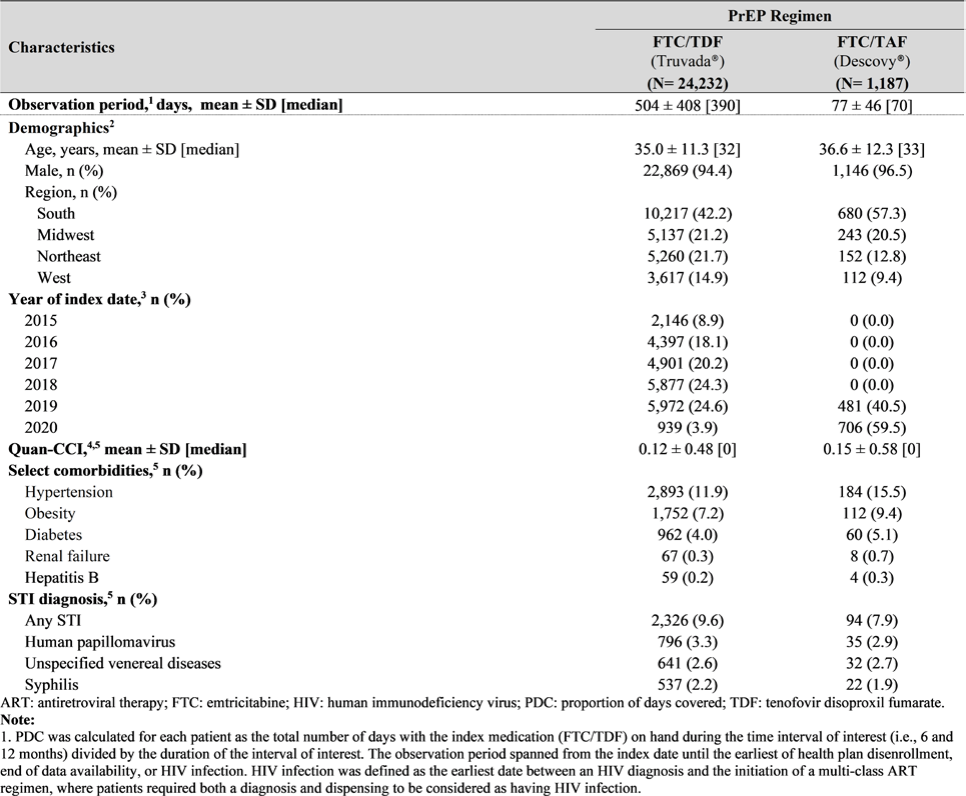

Figure 1. Proportion of Days Covered of FTC/TDF Users

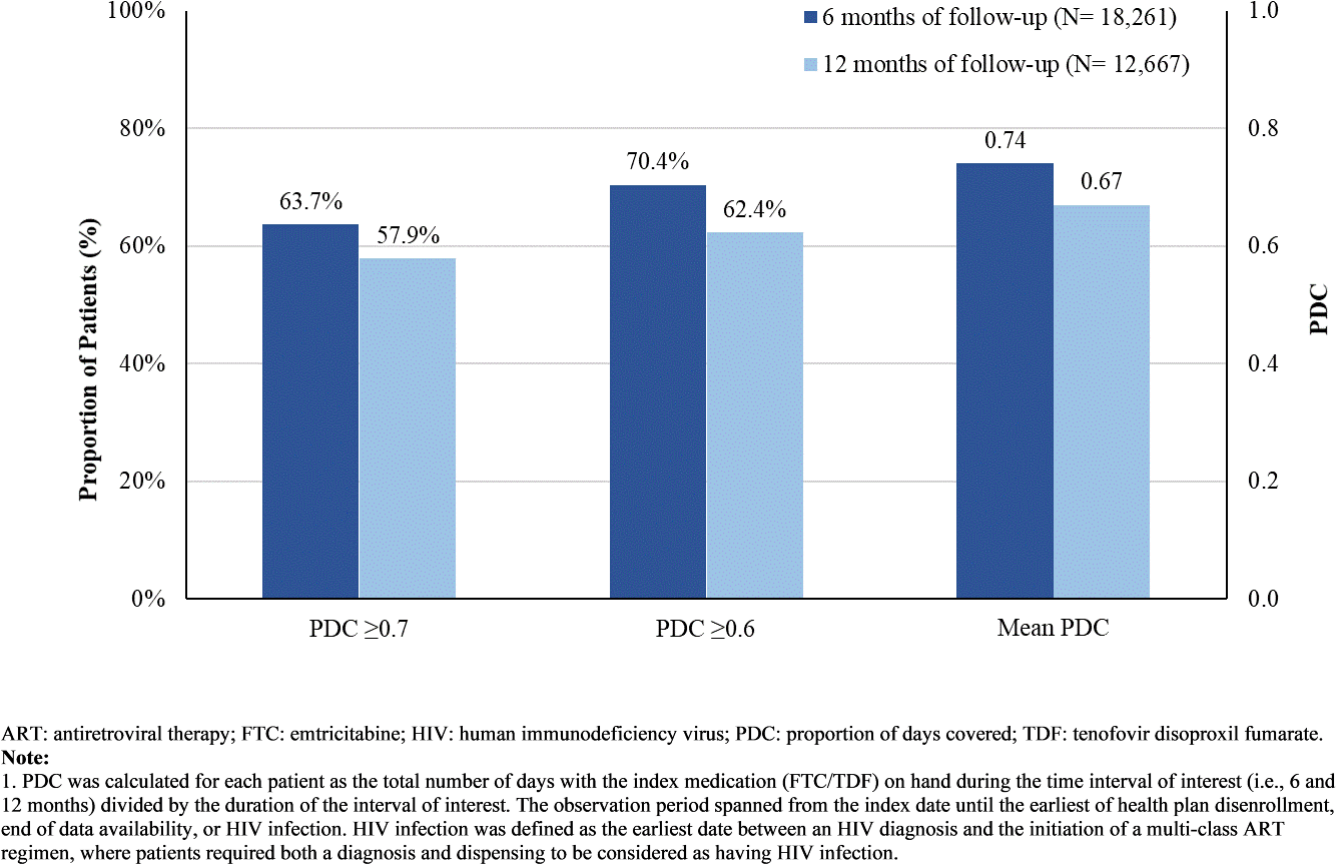

Figure 2. Kaplan-Meier Persistence Rates of FTC/TDF Users

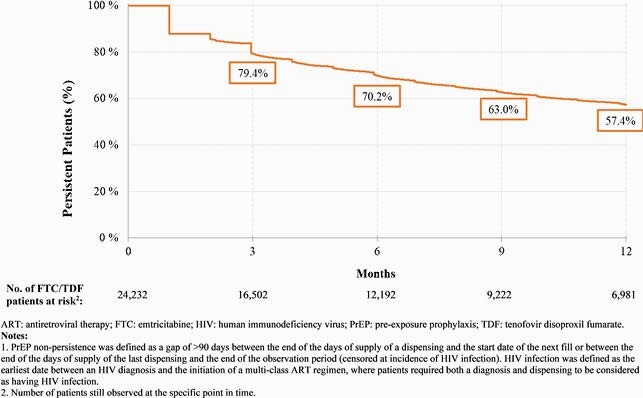

**Conclusion:**

Patient characteristics of PrEP users are broadly similar between regimens, though switching from FTC/TDF to FTC/TAF is common. FTC/TDF users had lower real-world PDC and persistence than in recent clinical trials (DISCOVER and HPTN 083).

**Disclosures:**

**Alan Oglesby, MPH**, **GlaxoSmithKline (GSK**) (Employee, Shareholder) **Guillaume Germain, MSc**, **ViiV Healthcare** (Other Financial or Material Support, I am an employee of Groupe d’analyse, Ltée, a consulting company that provided paid consulting services to ViiV Healthcare for the conduct of the present study.) **Francois Laliberte, MA**, **Viiv** (Research Grant or Support) **Staci Bush, NP**, **GlaxoSmithKline (GSK**) (Employee, Shareholder) **Heidi Swygard, MD**, **ViiV Healthcare** (Employee) **Sean MacKnight, MScPH**, **Analysis Group** (Employee) **Annalise Hilts, BA**, **Analysis Group, Inc.** (Employee) **Mei Sheng Duh, MPH, ScD**, **ViiV Healthcare** (Grant/Research Support)

